# A phase I trial of cyclosporine for hospitalized patients with COVID-19

**DOI:** 10.1172/jci.insight.155682

**Published:** 2022-06-08

**Authors:** Emily A. Blumberg, Julia Han Noll, Pablo Tebas, Joseph A. Fraietta, Ian Frank, Amy Marshall, Anne Chew, Elizabeth A. Veloso, Alison Carulli, Walter Rogal, Avery L. Gaymon, Aliza H. Schmidt, Tiffany Barnette, Renee Jurek, Rene Martins, Briana M. Hudson, Kalyan Chavda, Christina M. Bailey, Sarah E. Church, Hooman Noorchashm, Wei-Ting Hwang, Carl H. June, Elizabeth O. Hexner

**Affiliations:** 1 Department of Medicine, Division of Infectious Diseases,; 2 Center for Cellular Immunotherapies, and; 3 Department of Microbiology, Perelman School of Medicine, University of Pennsylvania, Philadelphia, Pennsylvania, USA.; 4 Parker Institute for Cancer Immunotherapy, University of Pennsylvania, Pennsylvania, USA.; 5 Department of Pharmacy, Hospital of the University of Pennsylvania, Philadelphia, Pennsylvania, USA.; 6 NanoString Technologies Inc., Seattle, Washington, USA.; 7 Yardley, Pennsylvania, USA.; 8 Department of Biostatistics, Epidemiology and Informatics and; 9 Department of Medicine, Division of Hematology, Perelman School of Medicine, University of Pennsylvania, Philadelphia, Pennsylvania USA.

**Keywords:** COVID-19, Chemokines, Cytokines

## Abstract

**BACKGROUND:**

COVID-19 remains a global health emergency with limited treatment options, lagging vaccine rates, and inadequate healthcare resources in the face of an ongoing calamity. The disease is characterized by immune dysregulation and cytokine storm. Cyclosporine A (CSA) is a calcineurin inhibitor that modulates cytokine production and may have direct antiviral properties against coronaviruses.

**METHODS:**

To test whether a short course of CSA was safe in patients with COVID-19, we treated 10 hospitalized, oxygen-requiring, noncritically ill patients with CSA (starting at a dose of 9 mg/kg/d). We evaluated patients for clinical response and adverse events, measured serum cytokines and chemokines associated with COVID-19 hyperinflammation, and conducted gene-expression analyses.

**RESULTS:**

Five participants experienced adverse events, none of which were serious; transaminitis was most common. No participant required intensive care unit–level care, and all patients were discharged alive. CSA treatment was associated with significant reductions in serum cytokines and chemokines important in COVID-19 hyperinflammation, including CXCL10. Following CSA administration, we also observed a significant reduction in type I IFN gene expression signatures and other transcriptional profiles associated with exacerbated hyperinflammation in the peripheral blood cells of these patients.

**CONCLUSION:**

Short courses of CSA appear safe and feasible in patients with COVID-19 who require oxygen and may be a useful adjunct in resource-limited health care settings.

**TRIAL REGISTRATION:**

This trial was registered on ClinicalTrials.gov (Investigational New Drug Application no. 149997; ClinicalTrials.gov NCT04412785).

**FUNDING:**

This study was internally funded by the Center for Cellular Immunotherapies.

## Introduction

SARS-CoV-2, the causative agent of COVID-19, is a novel coronavirus that induces an acute respiratory disease with systemic complications that range from minimally symptomatic, self-limited disease to critical illness and death. The COVID-19 pandemic is an ongoing global health crisis. Although vaccine development has been rapid, safe, and effective, treatment of disease has largely suffered from a paucity of effective antiviral drugs and variable impact of antiinflammatory agents, some of which are both costly and associated with long-term immunosuppression. Global infection rates continue to rise, vaccines are lagging, and case fatality rates vary globally but remain in the 1%–10% range. As of March 2022, global infections exceeded 420 million, with over 5.8 million deaths (1). Severe pneumonia occurs in approximately 15% of cases and drives mortality. Access to critical care resources, such as ventilators or even supplemental oxygen, remains an unmet need in some areas more than 2 years into the pandemic.

COVID-19 is characterized by immune dysregulation or cytokine storm, an orchestrated response involving infected cells, effector T cells, macrophages, and other innate immune cells as well as the cytokines/chemokines produced that collectively result in widespread lung inflammation (2). Hospitalized patients with severe COVID-19 exhibit high serum levels of type I IFN (IFN-I; IFN-α and IFN-β), type II IFN (IFN-II; IFN-γ), IL-2, IL-7, IL-10, granulocyte colony–stimulating factor, TNF, C-X-C motif chemokine ligand 10 (CXCL10), monocyte chemoattractant protein 1 (MCP1), and macrophage inflammatory protein (MIP1α) (3–5). Systemic elevations of these cytokines, C-reactive protein (CRP), and ferritin accompanied by lymphopenia are frequently observed in patients with COVID-19 and are also hallmarks of patients with hemophagocytic lymphohistiocytosis, also referred to as macrophage activation syndrome (HLH) (6).

Calcineurin inhibitors are a class of noncytotoxic immunosuppressive drugs that selectively impair T cell function by blocking nuclear factor of activated T cells (NFAT) signaling and downstream cytokine production. A drug screen conducted at the University of Pennsylvania identified cyclosporine as an active antiviral agent in human lung cells (7). Currently cyclosporine A (CSA), tacrolimus, and sirolimus are widely used to prevent rejection in solid organ transplant and for the treatment of arthritis and psoriasis; CSA is also the backbone of most protocols treating HLH. Given the shared inflammatory pathways seen in both HLH and the immune dysregulation seen in severe COVID-19 infection, we hypothesized that CSA could be an effective antiinflammatory agent for the treatment of COVID-19–associated immunopathology. Furthermore, CSA is not myelosuppressive and has also been shown to have direct antiviral effects by inhibiting coronavirus replication (8–10). Thus, we asked whether, if properly timed in patients with COVID-19, CSA would be sufficiently safe so that, ultimately, it could serve as a broad-spectrum inhibitor to help control SARS-CoV-2 infection, decrease severity of cytokine storms, and improve outcomes. This early intervention with a well-characterized and approved medication might be of particular importance in resource-poor or resource-limited areas.

## Results

### Patients.

**Figure 1:**
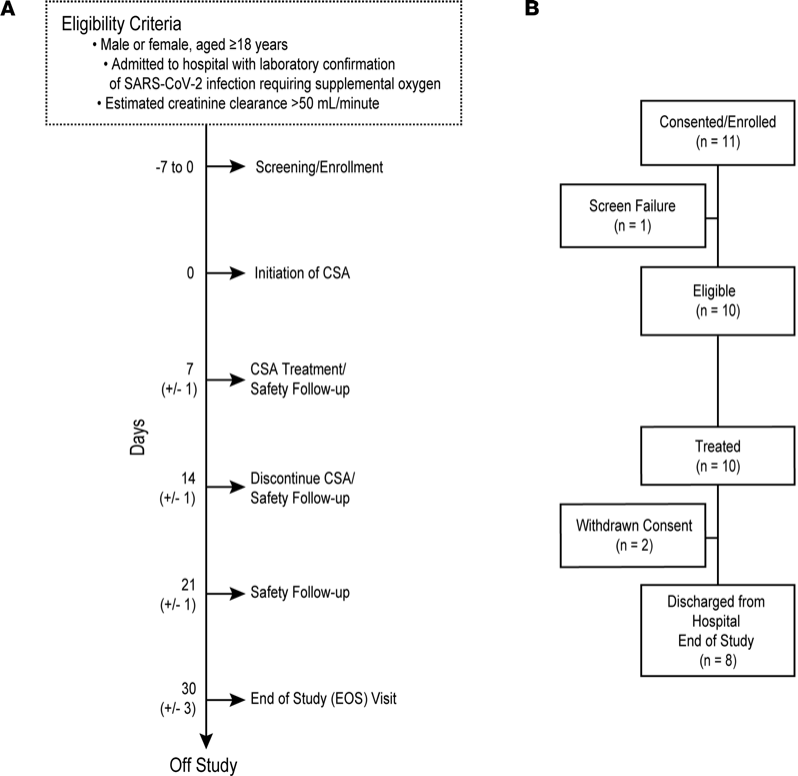
Protocol design and consort diagram for trial of CSA in hospitalized patients with COVID-19. (**A**) Eligibility criteria and protocol schema for screening, CSA treatment, and follow-up safety assessments. If hospital discharge occurred prior to day 14, treatment was discontinued at discharge. (**B**) CONSORT diagram indicating the number of patients screened and enrolled in the study.

Eleven patients consented to the study and 10 patients were treated (Figure 1). The median age was 57.5 years (Table 1). All patients required oxygen, with a median National Early Warning Score of 3 (score range, 2–8).

### Cyclosporine levels.

**Figure 2:**
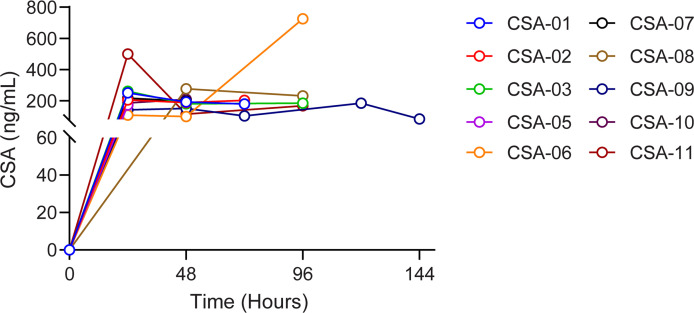
Serial trough levels of CSA following intervention. CSA was administered at a starting dose of 9 mg/kg/d and adjusted to target a trough level of 200–300 ng/mL. Each differently colored line represents CSA trough levels over time for an individual patient.

While the optimal dose is unknown for this indication, for this initial safety study a standard transplant target dose was selected to achieve a trough level of 200–300 ng/mL. All patients received only the oral capsule formulation of CSA. The median number of days of treatment was 4 (range, 2–6 days), and the median doses received was 8 (range, 3–11 doses). Trough levels (Figure 2) ranged from 83 ng/mL to more than 500 ng/mL. The target trough level was achieved in 80% of patients, and 80% of patients had dose modifications based on therapeutic drug monitoring.

### Concomitant treatments.

The trial opened on June 22, 2020, when remdesivir was still being investigated. Remdesivir gained approval during the course of the trial, and use of dexamethasone was introduced as part of our standard institutional treatment protocol. All patients received both remdesivir and dexamethasone as part of standard-of-care treatment.

### Safety.

Five patients (50%; CI, 22%–78%) experienced adverse events (AEs) (Table 2). Transaminitis was the most common AE, and there was 1 event each of headache and creatinine increase. Two patients discontinued treatment due to AEs. No patients required intensive care unit–level (ICU-level) care, and all patients were discharged from the hospital alive. There were no events of posterior reversible leuko-encephalopathy syndrome/reversible posterior leukoencephalopathy syndrome or microangiopathy, and no serious AEs.

### Reduction in pathogenic inflammation.

**Figure 3:**
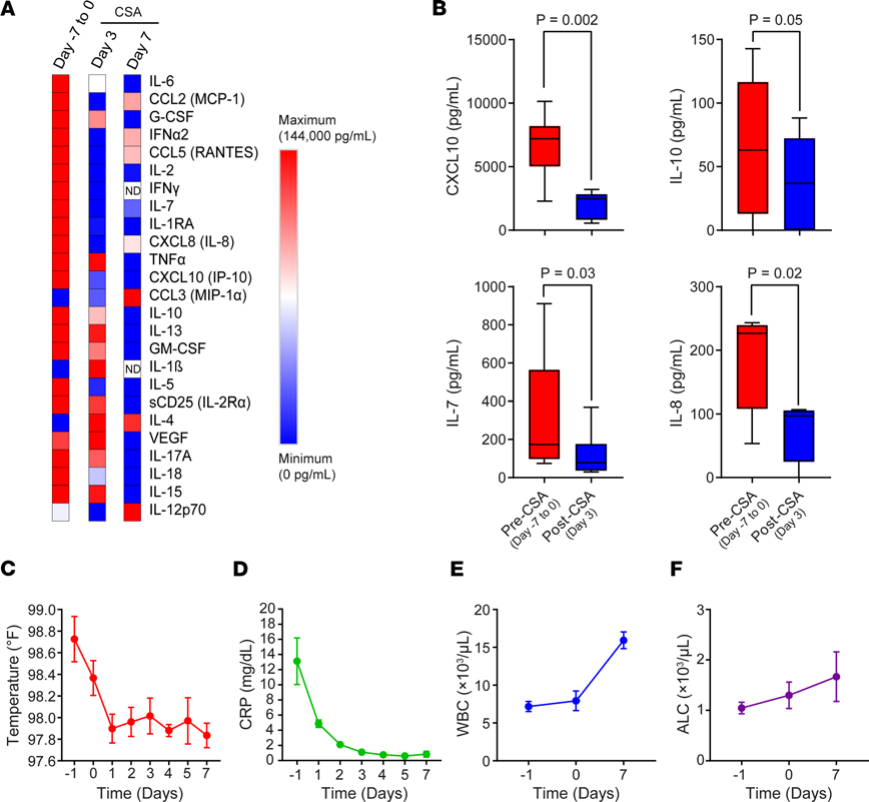
Characterization of the hyperinflammatory state in hospitalized patients with COVID-19 treated with CSA. (**A**) Heatmap showing changes in expression of several serum inflammatory cytokines/chemokines over time in hospitalized patients with COVID-19. ND, not detected. (**B**) Box plots depicting baseline and post–CSA treatment (days 3 and 7) serum levels of select proinflammatory mediators implicated in the COVID-19 cytokine storm in evaluable patients. The boxes depict the first and third quartiles and bands within boxes indicate medians. Maximum and minimum data points are depicted by whiskers. *P* values were calculated using a parametric 2-sided Student’s *t* test for paired samples. Clinical assessments, such as (**C**) body temperature, (**D**) inflammation status (CRP levels), (**E**) white blood cell (WBC) counts, and (**F**) absolute lymphocyte counts (ALC) are shown across all longitudinal time points for *n* = 10 patients with COVID-19 treated with CSA. Data are shown as the mean ± SEM.

On study entry, all patients met criteria for hyperinflammation based on COV-H criteria. Serum specimens were analyzed to quantify circulating proinflammatory cytokine/chemokine levels using multiplex bead–based immunoassays. In most of the 10 patients accrued (*n* = 6), samples were collected twice, typically from day –7 to 0 (baseline) and at day 3 after CSA administration. A subset of patients (*n* = 5) had cytokine measurements performed more than once after enrollment. At baseline, we detected high levels of multiple proinflammatory cytokines and chemokines known to characterize COVID-19–associated hyperinflammation (Figure 3A) (3). Significant or near significant reductions in CXCL10, IL-10, IL-7, and IL-8 were observed on day 3 following CSA administration, relative to pretreatment time points (Figure 3B). The overall inflammatory cytokine signature continued to decrease from day 3 to day 7 after CSA administration (Figure 3A), in association with reductions in body temperature (Figure 3C) and noncardiac CRP levels (Figure 3D). Reductions in proinflammatory cytokine production coincided with increased white blood cell (Figure 3E) and absolute lymphocyte counts (Figure 3F).

### Immune gene signatures of PBMCs from CSA-treated patients.

**Figure 4:**
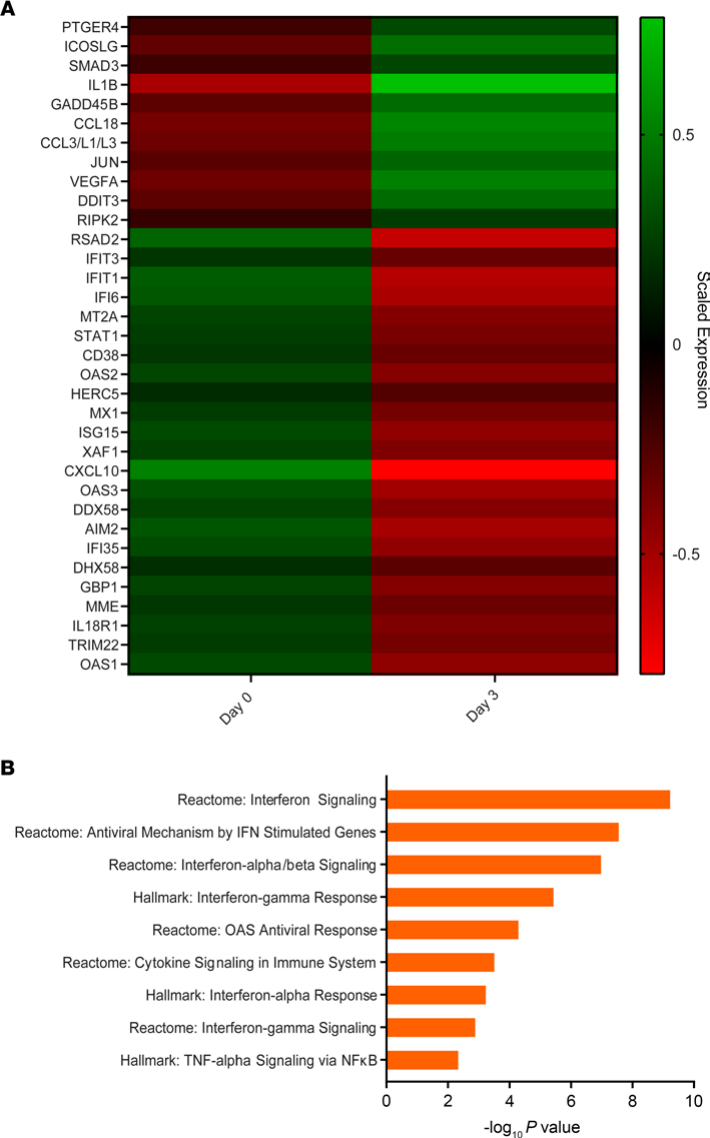
Gene expression profiles of PBMCs from CSA-treated patients. (**A**) The heatmap illustrates the expression differences of immune-related genes at baseline (day 0) and after CSA treatment (day 3). The map contains scaled expression levels that are color coded with red, corresponding to downregulation, and green, corresponding to upregulation. (**B**) Gene set enrichment analysis (GSEA) for immune-related genes is shown. GSEA was performed using pathways derived from gene sets belonging to the Molecular Signatures Database (i.e., Hallmark and Reactome gene sets).

To gain further insight into host responses to SARS-CoV-2 infection in the setting of CSA treatment, we measured the transcript levels of 773 genes associated with immune response dynamics using NanoString technology. We compared changes in gene expression in PBMCs from patients before CSA treatment and at day 3 after CSA administration (Table 3 and Figure 4A). Expression levels of several genes associated with the IFN response (e.g., *RSAD2, IFIT3, MT2A, STAT1, OAS2, MX1*) and innate immune cell activation (e.g., *CXCL10, MME, DDX58, IL18R1*) were downregulated following CSA treatment (Figure 4A). The top functional pathways associated with these transcriptional changes included those involved in IFN-I signaling, the IFN-γ response, and TNF-α signaling via NF-κB (Table 4 and Figure 4B). Altogether, these data highlight a predominance in the reduction of IFN gene expression profiles in blood cells following CSA treatment of patients with COVID-19.

## Discussion

CSA is approved by the US FDA for prophylaxis of organ rejection in solid organ transplant recipients as well as treatment of rheumatoid arthritis and severe psoriasis. CSA, given for a short course to hospitalized patients with COVID-19 who require oxygen, is feasible and appears safe. AEs were consistent with the known safety profile of CSA in other populations (10–12). Notably some of the AEs may have reflected either infection with SARS-CoV-2 and/or toxicity of other medications, including remdesivir. The majority of AEs were mild, and no stopping rules were met. Notably, all patients improved, and none required mechanical ventilation. This initial study was not designed to determine efficacy as to whether CSA affected outcomes. An ideal time point for intervention with CSA may be before potentially aberrant immune activation has occurred but after sufficient time has passed, allowing priming of the humoral immune response to SARS-CoV-2. This is in contrast to immunologically naive patients, because administration of CSA prior to infection can be deleterious (12).

One advantage to using CSA is that it is a cost-effective intervention, which could benefit resource-limited settings. Drug acquisition costs are low, and this medication is widely available in both pill and liquid formulations, making it easy to administer orally to diverse populations. It is likely that lower doses can be given without a need for therapeutic drug monitoring. Because remdesivir is a substrate for cytochrome P450 3A4 (CYO3A4), organic anion transporting polypeptide 1B1 (OATP1B1), and P-glycoprotein 1 (P-gp) in vitro (reviewed in refs. 13, 14), there is a potential for a significant drug interaction with CSA. However, based on our experience, this interaction appears to be manageable. Additionally, given the short recommended duration of therapy with remdesivir and the likelihood that short courses of cyclosporine will also be beneficial, we do not anticipate significant AEs related to the coadministration of these medications. Although we excluded individuals with creatinine clearances of less than 50 mL/min, in this proof-of-concept study, we had ample experience safely administering CSA to patients with reduced glomerular filtration rate (GFR) in the setting of transplantation. Thus, we believe that, with monitoring of renal function, CSA could be a viable option for administration in patients with COVID-19 and reduced GFR. At the initiation of our study, dexamethasone became the standard of care for patients experiencing hypoxia. Because we did not want to deprive patients of this intervention, we opted to add CSA to the treatment regimen. This could confound the effect and the effect size of each intervention independently. More experience with diverse populations will help further clarify this issue. Nevertheless, our study does provide initial safety information regarding concomitant use of these 2 immunosuppressive agents.

Several reports have revealed that the hyperinflammatory response associated with COVID-19 is a major cause of disease severity and death. In this study, we detected decreased levels of several proinflammatory cytokines/chemokines, including CXCL10, following CSA administration compared with levels at baseline time points. Continuously high levels of CXCL10 have been previously associated with increased viral load, loss of respiratory function, lung injury, and a fatal outcome in SARS-CoV-2 infection (14). Furthermore, it has been proposed that CXCL10 may mediate the aberrant immune response that controls the duration of mechanical ventilation in patients with COVID-19 with acute respiratory distress syndrome (ARDS) (15). CXCL10 has also been associated with disease severity in H5N1, H1N1, SARS-CoV, and MERS-CoV (16–19). Notably, CSA is a robust inhibitor of CXCL10-induced NFATc1 activation, a mechanism by which CXCL10 regulates the recruitment of inflammatory cells in rheumatoid arthritis (20). Thus, modulation of CXCL10 through short-course CSA treatment may be a promising therapeutic approach to prevent progression to COVID-19 related ARDS.

We also showed downregulation of IFN-I– and IFN-II–driven hyperinflammatory gene expression profiles in peripheral blood cells of patients with COVID-19 following CSA administration. Notwithstanding the results of early studies to the contrary, emerging evidence suggests that a robust IFN-I response occurs in severe SARS-CoV-2 infection (14, 21, 22), which contrasts with a delayed, potentially diminished, IFN response observed early during infection (23, 24). A strong IFN-I response could exacerbate hyperinflammation in the context of severe COVID-19 disease progression through several different mechanisms, including abrogation of the tolerizing effects of TNF and potentiation of increased monocyte and macrophage responsiveness to additional Toll-like receptor signals (25). CSA blocks the release of mitochondrial factors that stimulate the production of IFN-I by innate immune cells (reviewed in ref. 26). Further understanding of the roles of various inflammatory and antiviral cytokines as well as chemokines at different stages of infection and in patients with mild versus severe SARS-CoV-2 infection will help elucidate the optimal therapeutic window for CSA in target subgroups of patients with COVID-19. Finally, it is worth noting that CSA may have direct antiviral effects, as it inhibits coronaviruses from binding to cyclophilin (7, 9), a critical step in the replication process.

This study has several limitations. Our enrollment was affected by the variable epidemiology of COVID-19 and the changing therapeutics during our study period; therefore, the number of patients studied was small. The low number of patients studied also affected our ability to estimate whether there was a significant clinical effect for our patients, independently of those related to corticosteroids and remdesivir. However, the complexities of clinical trial execution in the COVID-19 era did not influence the design or the foundational aspects of this study. The trial was conducted to determine the initial safety of using an immunosuppressive medication in a seriously ill, infected population, prior to considering potential subsequent comparative clinical investigations to determine efficacy.

In summary, in this proof-of-concept study, we have shown that CSA is a safe and potentially effective therapeutic intervention for patients with SARS-CoV-2 infection. Its antiinflammatory properties, wide availability, safety, and low cost make it a particularly attractive modality for use in resource-limited settings. A prospective randomized controlled trial testing the efficacy of cyclosporine for the treatment of COVID-19 pneumonia is registered in Spain (27). Based on our results further large-scale trials are warranted to explore the safety and benefits of this intervention globally.

## Methods

### Design and study population.

This was a phase I single-site, single-arm, open-label study of a short course CSA treatment of hospitalized patients with COVID-19. Eligible patients were adults over the age of 18 years, admitted to hospital with laboratory-confirmed SARS-CoV-2 infection and requiring supplemental oxygen, with an estimated creatinine clearance of more than 50 mL/min. Patients were excluded if they were admitted to the ICU at time of enrollment; they had an additional, active uncontrolled infection with a non–COVID-19 pathogen; they had an active malignancy; or they were on chronic immunosuppressive medications for other indications. They were also excluded if they had received prior treatment with immunomodulators or immunosuppressant drugs within 5 half-lives or 30 days of consent, such as IL-1, IL-6, or TNF inhibitors or Janus kinase inhibitors. Also excluded were pregnant or lactating women and patients receiving investigational vaccines for SARS-CoV-2. Dexamethasone as standard-of-care therapy for SARS-CoV-2 and use of inhaled steroids were allowable.

### Intervention.

The study treatment, cyclosporine (modified, Gengraf) capsules (25 and 100 mg) were given at an initial starting dose of 9 mg/kg/d orally, which was divided into 2 doses and given every 12 hours, with a maximum dose of 400 mg/dose for all participants. An oral solution (100 mg/mL) or intravenous formulation (Sandimmune; 3 mg/kg/d given by continuous infusion) was also available for patients unable to swallow capsules. Therapeutic drug monitoring was performed on day 2 and every Monday, Wednesday, and Friday during active dosing. Subsequent cyclosporine dosing was adjusted to target a trough level of 200–300 ng/mL without a maximum dose level. The intended duration of administration was up to 14 days with planned treatment discontinuation if mechanical ventilation was required. Treatment was held for marked elevations in creatinine or transaminases and discontinued for all patients at the time of hospital discharge.

### Outcomes.

This was a study to assess the safety of CSA in patients with COVID-19, as measured by treatment-related AEs on study ICU transfer, secondary infections, and need for mechanical ventilation or increase in supplemental oxygen requirements. Samples for exploratory measurements (e.g., serum cytokine levels) were also collected.

This safety study also defined the following events as ones that would trigger a pause of the study: moderate-to-severe superinfection; severe microangiopathy or posterior reversible leuko-encephalopathy syndrome, also known as reversible posterior leukoencephalopathy syndrome; and patient death.

### Laboratory and correlative analyses.

Clinical laboratory tests at screening/enrollment and after CSA administration included complete blood counts and assessment of CRP levels and ferritin levels. We used the CRP and ferritin levels to determine whether patients exhibited hyperinflammation using COV-H criteria (28). Routine blood analysis was performed using a fully automated cell counter in the University of Pennsylvania Hematology Laboratory. CSA concentration was measured in whole blood samples using a validated liquid chromatography–tandem mass spectrometry method (29).

For cytokine measurements, serum samples stored at –80°C were thawed and centrifuged at 2000*g* for 5 minutes. The LEGENDplex COVID-19 Cytokine Storm Panels 1 and 2 (BioLegend) were used to determine cytokine concentrations, with sample analysis performed using an LSRFortessa flow cytometer (BD Biosciences). Serum was diluted 1:2 and the procedure was carried out according to the manufacturer’s instructions, with the exception that capture beads were inactivated in 4% paraformaldehyde for 15 minutes at room temperature and washed once in wash buffer after completion of the staining protocol in a class II biosafety cabinet under BSL-2^+^ conditions.

### Gene expression analyses.

Total RNA was extracted from patient PBMCs using the Qiagen RNeasy Plus Mini Kit as per the manufacturer’s instructions (Qiagen). The eluted RNA (20 μl) was stored at −80°C until further use.

The nCounter assays were carried out using the NanoString nCounter Analysis System (NanoString Technologies). Hybridization reactions were performed according to the manufacturer’s instructions. nCounter Immune exhaustion and Host Response containing a biotinylated capture probe for target genes and housekeeping genes and reporter probes attached to color-coded barcode tags were hybridized to 200 ng of total RNA for 18 hours at 65°C. Samples were processed using an automated nCounter Prep Station. Hybridized samples were purified and immobilized in a sample cartridge for data collection, followed by the quantification of target mRNA in each sample using the nCounter Digital Analyzer.

Quantified expression data were analyzed by ROSALIND (https://rosalind.bio/), with a HyperScale architecture developed by ROSALIND. Read distribution percentages, violin plots, identity heatmaps, and sample MDS plots were generated as part of the QC step. Normalization, fold changes, and *P* values were calculated using criteria provided by Nanostring. ROSALIND follows the nCounter Advanced Analysis protocol of dividing counts within a lane by the geometric mean of the normalizer probes from the same lane. Housekeeping probes to be used for normalization are selected based on the geNorm algorithm as implemented in the NormqPCR R library (30). Fold changes and *P* values are calculated using the fast method as described in the nCounter Advanced Analysis 2.0 User Manual. *P* value adjustment is performed using the Benjamini-Hochberg method of estimating FDRs. Clustering of genes for the final heatmaps of differentially expressed genes was done using the PAM (partitioning around medoids) method which incorporates the fpc R library (31) that takes into consideration the direction and type of all signals on a pathway, the position, role, and type of every gene, etc. Hypergeometric distribution was used to analyze the enrichment of pathways, gene ontology, domain structure, and other ontologies. The topGO R library (32) was used to determine local similarities and dependencies between gene ontology terms in order to perform Elim pruning correction. Several database sources were referenced for enrichment analysis, including MSigDB (33, 34) and REACTOME (35). Enrichment was calculated relative to a set of background genes relevant for the experiment.

### Statistics.

The initial trial was designed to enroll approximately 25 patients, with a target of 20 patients maximum treated with CSA. We estimated that 20% of enrolled patients would not receive CSA, primarily owing to unanticipated rapid clinical deterioration or improvement. The sample size enabled us to provide reasonable precision for rates of AEs; at a sample size of 20, the half-width of the 90% CI for an AE rate would be no more than 20%. After study activation, accrual rate tracked with infection rates; when infection rates were declining, lengths of hospital stays were decreasing. When vaccines became widely available a decision was made to close the study. With a reduced sample size of 10 patients, we have probability of 0.9 that the true AE rate would be less than 26% if no AEs were observed and less than 40% if 1 AE was observed. Descriptive statistics were computed for all endpoints.

Serum cytokine levels were analyzed between time points using 2-tailed paired Student’s *t* tests. Statistical analyses were performed using Prism 8 GraphPad. *P* values of less than 0.05 were considered to be statistically significant.

### Study approval.

This investigator-initiated trial protocol was approved by the Institutional Review Board at the University of Pennsylvania and was overseen by the Center for Cellular Immunotherapies (IND no. 149997, ClinicalTrials.gov NCT04412785). Informed consent was obtained in person from each patient by a physician investigator, and the consent was signed and documented in the medical record.

## Author contributions

EAB, PT, JAF, IF, AM, WR, HN, WTH, CHJ, and EOH participated in study design. EAB, PT, AM, A Chew, EAV, A Carulli, ALG, AHS, TB, RJ, and RM participated in patient recruitment and data collection. JHN, JAF, BMH, KC, CMB, and SEC conducted laboratory experiments. EAB, JAF, WTH, CHJ, and EOH analyzed the data. EAB, JAF, and EOH wrote the first draft of the manuscript, which was then reviewed and revised by all authors.

## Supplementary Material

ICMJE disclosure forms

## Figures and Tables

**Table 4:**
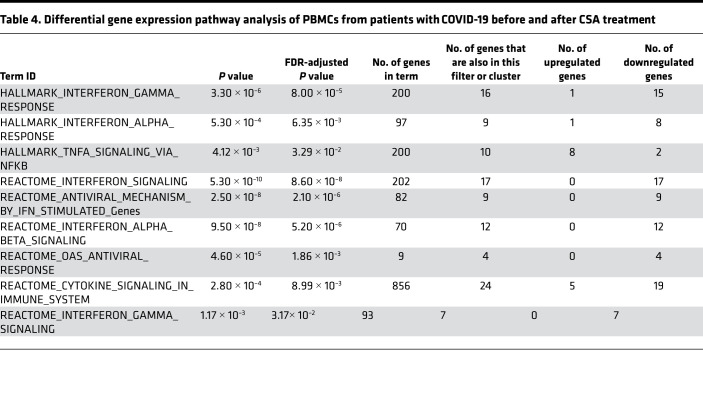
Differential gene expression pathway analysis of PBMCs from patients with COVID-19 before and after CSA treatment

**Table 3:**
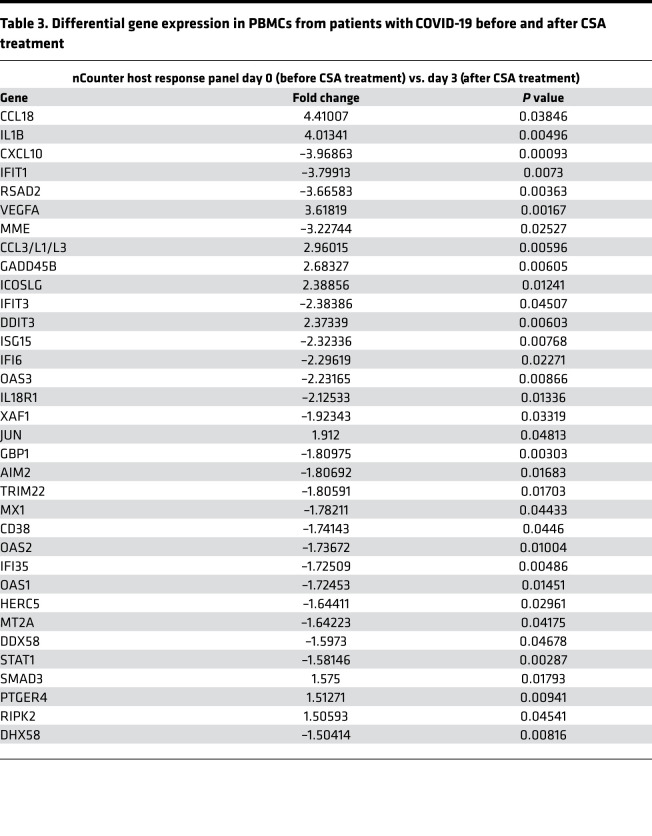
Differential gene expression in PBMCs from patients with COVID-19 before and after CSA treatment

**Table 2:**
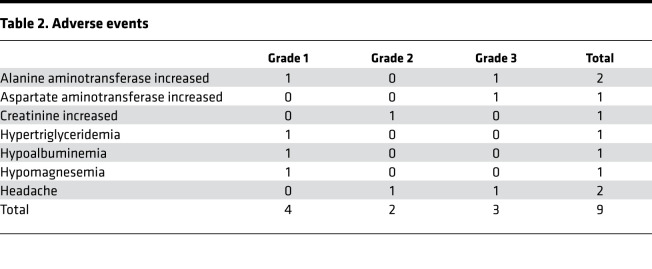
Adverse events

**Table 1:**
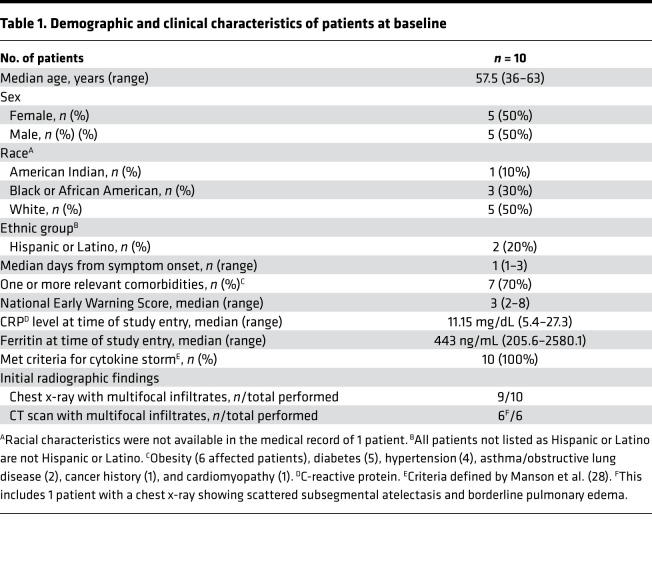
Demographic and clinical characteristics of patients at baseline

## Version 1 05/10/2022

In-Press Preview

## Version 2 06/08/2022

Electronic publication
